# Hematologic parameters in female alpacas during age progression: a retrospective study

**DOI:** 10.1038/s41598-023-50572-9

**Published:** 2024-01-04

**Authors:** Matthias Gerhard Wagener, Max Kornblum, Frederik Kiene, Martin Ganter, Ulrike Teichmann

**Affiliations:** 1 Clinic for Swine and Small Ruminants, Forensic Medicine and Ambulatory Service, University of Veterinary Medicine Hannover, Foundation, Hannover, Germany; 2 Max Planck Institute for Multidisciplinary Sciences, Göttingen, Germany

**Keywords:** Animal physiology, Ageing

## Abstract

Alpacas, like all camelids, have elliptical red blood cells (RBCs) in contrast to other mammals. This particular shape is important for increased osmotic resistance and stability. Age-related changes in the RBC count are known in other species, with alterations in both red and white blood cells being described. In alpacas, there are few data on age-related changes, and only a comparison of crias with adult animals. We characterized age-related hematologic changes in a study of 21 female alpacas from a research herd. A total of 87 records of clinically healthy alpacas of different ages were statistically analyzed retrospectively from the hematologic records over a nine-year period. Significant positive correlations of age with hemoglobin (Hb), HCT, MCV, MCH, neutrophils, platelet-to-lymphocyte ratio (PLR), and neutrophil-to-lymphocyte ratio (NLR) were found as well as significant negative correlations of age with lymphocytes in addition to lymphocyte-to-monocyte ratio (LMR). A paired comparison of eight older animals in the herd at three different ages also showed significant differences in the parameters Hb, HCT, MCV, MCH, MCHC, lymphocytes, eosinophils and neutrophils. Similar changes in hematologic parameters have been reported in other species and should be taken into account when interpreting hematologic results in alpacas.

## Introduction

Alpacas are becoming increasingly popular as hobby animals in Europe^1–3^. Common diseases include disorders of the gastrointestinal tract, the respiratory tract, the skin, liver, and teeth^4–6^. In case of disease, the animals often show clinical signs only at a late stage. Hematologic diagnostics are therefore of great importance in the veterinary management of South American camelids (SACs)^7^.

Like other camelids, alpacas have an erythrocyte (RBC: red blood cell) morphology that is unusual for mammals. Instead of having a round silhouette, their RBCs are elliptical^8^. Due to their cytoskeletal network and membrane proteins, camelid RBCs have a greater stability and osmotic resistance than those of other species^9–11^. Furthermore, unlike those of other mammalian species, camelid RBCs do not deform^12^. This higher stability of the RBCs is necessary in camelids because they have to tolerate large fluctuations in the animal's water balance^13^. These characteristics may influence the results of automated hematology tests, as hematocrit (HCT) is often determined by calculation from MCV and RBC count rather than by microhematocrit centrifugation^14–17^. Similarly to goats and in contrast to some ruminants such as cattle, sheep, or deer, alpacas and other SACs have rather small RBCs^17–22^.

A moderate number of scientific publications provide reference intervals (RI) for the assessment of hematologic findings in alpacas^19,20,23–28^. Some of the sources distinguish between male and female animals^19,27,28^. With regard to age, only two studies provide separate RI for crias (young alpacas < 6 months of age) and adult alpacas^19,27^. Hengrave-Burri et al.^19^ found significant differences between crias and adult alpacas for hematologic RI of the parameters MCV and absolute numbers of eosinophils and lymphocytes. Furthermore, significant differences between the sexes for hemoglobin (Hb) and RBCs were observed. Females had lower hemoglobin and RBC values than males, crias had lower values for MCV and eosinophils compared to adults and higher lymphocytes^19^. Hengrave-Burri et al. also studied the hematology of llamas and came to similar conclusions regarding sex and age. They also found significant differences between alpacas and llamas for most hematologic parameters^19^. The latter study, which examined the hematologic findings of alpacas in relation to sex and age was conducted by Husakova et al.^27^ and published in 2015. Females had lower eosinophils and higher lymphocytes than males. Age effects were observed, as crias had a significantly higher RBC count and calculated MCHC compared with adults, and significantly lower values for MCV, MCH, total leukocytes (WBC: white blood cell), neutrophils, eosinophils, and lymphocytes^27^. In addition to sex and age differences, seasonal differences in hematologic findings in alpacas were also evident in this study. Both Hengrave-Burri et al.^19^ and Husakova et al.^27^ concluded from their studies that sex and age should be considered when interpreting hematologic values in alpacas. However, in both studies comparison was only made between crias (< 6 months) and adult alpacas (> 6 months)^19,27^.

To the best of our knowledge, there are no data on the influence of age progression on hematologic parameters in adult alpacas. In order to gain an overview of the physiologic hematologic changes in animals over one year of age, and to assess the need to establish age-based RI for alpacas, we retrospectively evaluated the hematologic findings of an alpaca herd, which is used for scientific research purposes.

## Material and methods

We divided the investigation into two studies: For study 1, we examined hematologic records from several years of each animal’s life, plotted the data descriptively by age in years, and performed a correlation analysis with age in days.

Since data were available for several years for individual animals, age progression could also be followed in individual animals. For study 2, we therefore divided the hematologic records of the eight oldest animals in the herd into three age groups:

- Young (between 1 and 3 years of age).
- Middle (between 4 and 6 years of age).
- Old (between 7 and 11 years of age).

This allowed us to follow the age progression of the individual animal through repeated measurements.

### Herd

The retrospective studies were carried out on a herd of 22 alpacas in northern Germany. The herd started out in 2013 with six females from the same breeder (breeder 1). In 2015, four females were obtained from another breeder (breeder 2). One of these females was pregnant and a male cria was born from this dam in 2016 and neutered in 2017. Further females were obtained from breeder 2 in 2019 (n = 9), in 2020 (n = 2), and in 2022 (n = 2). According to the breeders, five of the animals (animal 3, 4, 10, 18, 19) were not purebred alpacas, but hybrids with llamas. In the case of one of these animals (#3), only one parent was an alpaca (50% llama), two of the animals (#10; #19) were the offspring of a hybrid and an alpaca (25% llama), in two other animals (#4; #18) the previous owner said that they were alpaca, but the phenotype of these animals suggested that there was also a hybrid component. All the other animals were definitely alpacas. Two animals in the herd died in the meantime. One animal that was integrated into the herd in 2015 had to be euthanized in 2019 at the age of five years due to hepatic lipidosis. Another animal integrated in 2019 was euthanized in 2020 at the age of two years. The animal was diagnosed with regenerative anemia, hypophosphatemia, copper deficiency, endoparasitosis, and *Candidatus* Mycoplasma haemolamae infection. Due to another case of disease in the herd in 2020 (animal #14 suffered from severe anemia and was only included in the evaluation after full recovery), some blood samples from the animals were tested positive in PCR for the presence of *Candidatus* M. haemolamae. This affected only 10 records (11.5%) from study 1. None of the animals showed any clinical signs of disease. As the pathogens infects RBCs^8^, it could be assumed that this could have an effect on RBC parameters, as it has been reported for hemotrophic mycoplasms in other species^29^. Although Viesselmann et al.^30^ found no differences in RBC counts, Hb, and HCT between healthy SAC-infected and non-infected animals with *Candidatus* M. haemolamae, Tornquist et al.^31^ reported an association of anemia with this pathogen in SACs in Peru^42^. To exclude an influence on the hematologic records, we compared hematologic records that were positive (n = 10; animals/year: 1/1;4/8;7/2;8/2;9/2;11/6;17/1;18/9;21/1;22/ < 1) with those that were negative for *Candidatus* M. haemolamae (n = 5; animals/year: 3/9;5/5;10/6;13/2;16/5). Hematologic parameters were compared by the t-test or Mann–Whitney U test, depending on distribution of data as evaluated by the Shapiro–Wilk test. None of the parameters was significant in the t-test or Mann–Whitney U test. Therefore, we assume that there was no significant effect of *Candidatus* M. haemolamae infection on the hematologic data in the animals studied.

The alpacas were housed in a stable and had permanent free access to a pasture. Water and hay were provided ad libitum. In addition, the animals were fed 320 g/animal/day of a concentrate for alpacas (Marstall® Alpaka & Co., Marstall Premium-Pferdefutter, Oberstaufen, Germany), which was divided into the morning and evening meal. Furthermore, the animals were fed 20 g/animal/day of an alpaca mineral feed (LAPAKO PRO MINERAL—Mineralfutter für Alpakas und Lamas, Migocki Tierernährung, Augsburg, Germany). From August 2019 to April 2020, the alpacas were also fed 17 g/animal/day of a concentrate with a higher zinc content (LAPAKO PRO ZINK für Alpakas/Lamas, Migocki Tierernährung) due to zinc deficiency in some of the animals. However, this does not appear to be a clinically relevant finding, as plasma zinc levels in alpacas are highly variable even under physiologic conditions^32^. In addition, the entire herd was fed small amounts of apples and carrots throughout the day. Animal feces were removed from the pasture daily. Every four to eight weeks, a pooled sample of feces from the herd was subjected to parasitological testing (flotation) to detect any sudden increase in worm eggs. Nevertheless, no sudden increase was detected during the study period. Deworming with regularly changing anthelmintics (doramectin, ivermectin, moxidectin, levamisole, fenbendazole, triclabendazole) took place once a year in summer after examination of fecal samples collected over three days. The success rate of deworming was usually assessed two weeks after administering the anthelmintic drug.

The alpaca herd is used by the Max Planck Institute for Multidisciplinary Sciences, Göttingen, Germany for biomedical research purposes, including immunization of the animals and subsequent collection of blood samples for the production of nanobodies. The animal facility is registered in accordance with §11 paragraph 1 TierSchG (Tierschutzgesetz der Bundesrepublik Deutschland, Animal Welfare Law of the Federal Republic of Germany) as documented by 39 20 00_2a) Si/rö , dated December 11, 2013 ("Erlaubnis, Wirbeltiere zur Versuchszwecken zu züchten und zu halten", "Permission to breed and to keep vertebrates for Experimental Purposes") by the Veterinär- und Verbraucherschutzamt für den Landkreis und die Stadt Göttingen (Veterinary and Consumer Protection Office for the district and city of Göttingen). Only a few animals are involved in an animal experiment at any one time. To monitor the health status of the herd, laboratory diagnostic blood tests were carried out on all animals at irregular intervals. These checks were usually carried out in the summer (69 between June and August of the 87 animals included in this study), but occasionally at other times of the year. For hematologic tests, EDTA blood samples (3–5 mL, S-Monovette K3 EDTA, Sarstedt) were obtained by puncturing a jugular vein of the animals. Blood smears were prepared from whole blood immediately after collection. EDTA blood and unstained blood smears were sent to an external commercial laboratory (SYNLAB.vet GmbH, Augsburg, Germany) for further diagnosis. The laboratory where the analyses were performed was accredited by the “Deutsche Akkreditierungsstelle GmbH”, certificate D-PL-14016-01-00 in accordance with DIN EN ISO/IEC 17025:2018 in 2020. The maximum interval between blood sampling and analysis was three days; on average, the hematologic data included in the analysis were measured after 1.29 ± 0.69 days (mean ± standard deviation) after blood sampling. Blood samples were analyzed using a widely used hematology analyzer (ADVIA 2120i, Siemens Healthcare GmbH, Erlangen, Germany). WBC measurements are based on flow cytometry and light scatter, differential WBC lysis, and myeloperoxidase staining^33^. The RBC indices are measured immediately after sphering RBCs^34^. HCT is calculated from MCV and RBC count (HCT = MCV × RBC count / 1000). Hemoglobin is determined colorimetrically using a cyanide-free reagent. A detailed overview of the analyzer's methods can be found in Harris et al.^33^. The settings used in the analyzer were a specific setting for alpacas provided to the laboratory by the manufacturer. A species-specific scattergram was evaluated based on the settings for horses. Regular maintenance and quality checks were carried out, including the measurement of a control sample at least twice a day (midday and evening), regular staff training, and regular inter-laboratory comparisons. For some of the blood samples (31 of 87 included in this study), microscopic examination of a blood smear prepared at the time of blood collection was performed. Blood smears were stained in the laboratory according to Pappenheim, and WBC differentiation was performed by experienced examiners differentiating 100 cells.

### Data collection

The laboratory data for each animal were available electronically as PDF files. A period of nine years (2013–2022) was evaluated. The results were transferred to an Excel spreadsheet in which each individual PDF file was entered as a separate column; the individual parameters from the laboratory results were entered as lines. In addition, the animal number, animal name, date of birth, date of sample collection, date of sample examination, and information on previous disease events, or actions taken in the context of other studies were entered as additional data as single lines in each column. The age of the animals for each sample was determined by calculating the difference between the date of birth and the date of sample collection and was expressed as both age in days and age in years. Several persons with many years of experience in veterinary hematology and SACs were involved in the design, conduct, and interpretation of the study.

### Inclusion and exclusion criteria for evaluation

**Figure 1:**
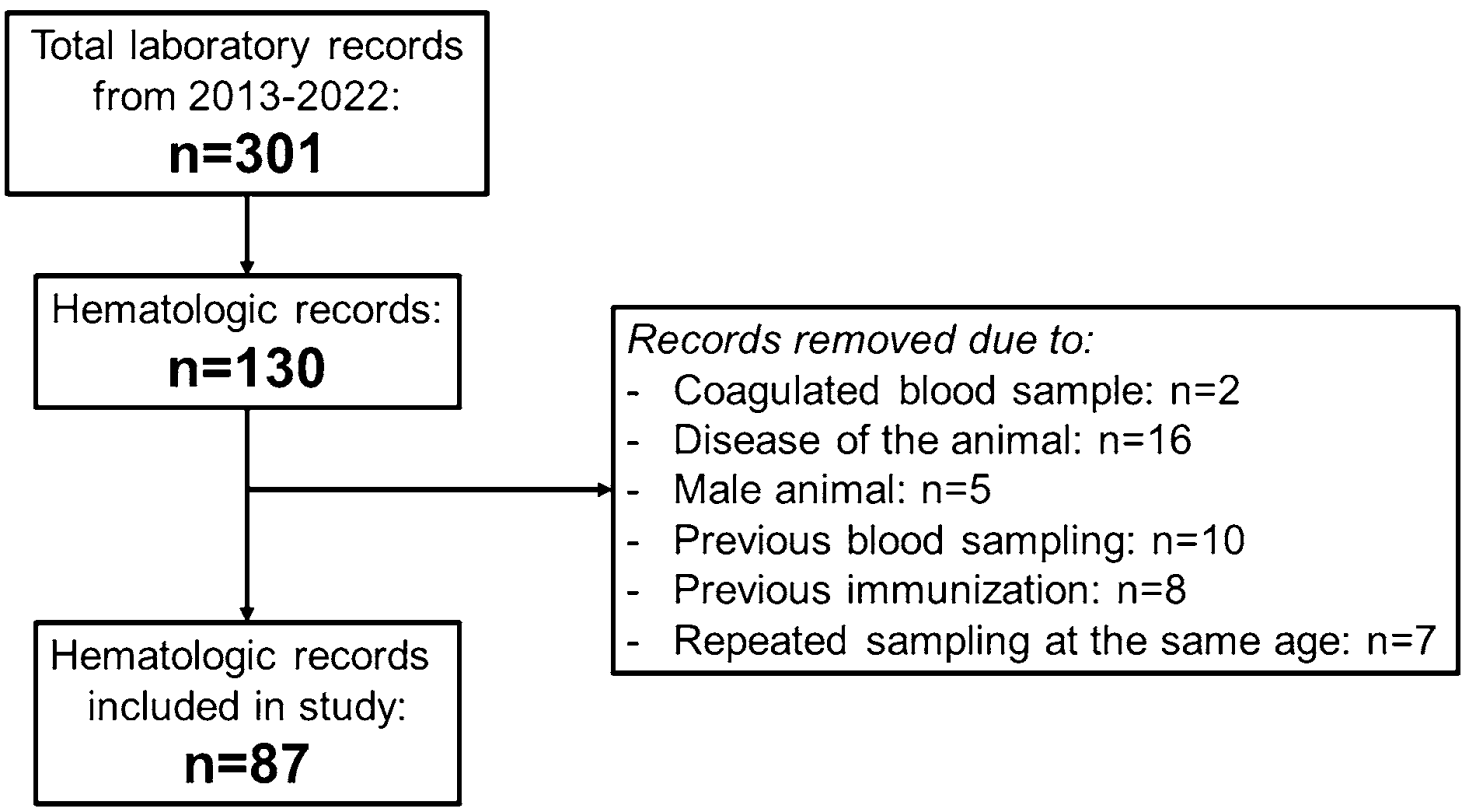
Flowchart of the selection process of the evaluated hematologic records from healthy female alpacas. The interval between an animal’s previous disease, previous immunization, or previous blood sampling and the hematologic record being considered from a healthy animal was at least 30 days. More than one exclusion criterion was applied to some hematologic records.

Some of the reviewed hematologic records had to be excluded after evaluation: Blood samples collected during or < 30 days after a documented disease event, immunization, or experimental blood collection procedure of the respective animal were excluded from the analysis. One animal (animal #12) was also excluded because it was the only male alpaca in the herd. Only one hematologic examination was evaluated from each animal per year of life to minimize bias due to over-representing. If more than one hematologic record met the inclusion criteria for evaluation in an animal during a year of life, only the sample with the greatest time interval after a documented disease event, immunization, or experimental blood collection procedure in that animal was included. More detailed information on the selection process can be found in the flowchart in Fig. 1 and in Supplementary Table S1.

### Evaluated parameters

Twenty-two parameters were reported in all of the selected hematology reports and were therefore available for evaluation. An overview of the parameters and their units and measurement methods is given in Supplementary Table S2. As different units are commonly used in different regions, we adopted the suggestions of Brereton et al. regarding the use of hematologic units^35^.

For our evaluation, the MCHC was expressed in [g/L] by multiplying the results in [g/dL] by a factor of 10. Furthermore, the absolute numbers of neutrophils, lymphocytes, monocytes, eosinophils, and basophils were expressed in [× 10^9^/L]. For this purpose, the values expressed in the unit [1/µL] provided by the laboratory were divided by 1000. The parameters "band neutrophils" and "other cells" were not evaluated because only one single band neutrophil was reported in only one blood sample (animal #16 at seven years of age), "other cells" were not found in any of the blood counts evaluated. Three additional hematologic parameters were calculated from the available data and included in the evaluation. These were:

- Neutrophil-to-lymphocyte ratio (NLR), which was calculated as follows: (segmented neutrophils [%] + band neutrophils [%]) / lymphocytes [%]
- Platelet-to-lymphocyte ratio (PLR), which was calculated as follows: platelets [× 10^9^/L] / lymphocytes [× 10^9^/L]
- Lymphocyte-to-monocyte ratio (LMR), which was calculated as follows: lymphocytes [%] / monocytes [%].

The LMR could not be determined in the records where monocytes were 0%. Therefore, a smaller number of records could be evaluated for the LMR than for the other parameters.

#### Albumin concentration

As the hydration status of an animal has an influence on hematologic parameters, this was also checked. Dehydration leads to an increase in HCT and albumin^36^. In addition to the hematologic parameters, the medical records at each sampling time included subsets of several biochemical parameters. In 83 of the 87 samples, albumin was determined from a serum sample collected in parallel. These measurements were performed photometrically using Beckmann Coulter AU5800 for samples until July 2020 and Abbott Alinitys thereafter.

### Statistical analysis

Statistical analysis of the data was performed using SAS Enterprise Guide 7.1 and R (R Foundation for Statistical Computing, Vienna, Austria, https://www.R- project.org) in combination with RStudio (Integrated Development for RStudio, Inc., http://www.rstudio.com).

### Study 1

Data were expressed as descriptive statistics with mean and standard deviation (SD), median, lower and upper quartile as well as minimum and maximum for each year of life. Spearman’s rank correlation coefficient was performed with the age of the respective animal in days and the hematologic parameters. The Spearman’s rank correlation coefficient was chosen because the age in days did not reveal normal distribution for all 87 hematologic records (Shapiro–Wilk test: p < 0.0001). A p-value less than 0.05 was considered significant (* = p < 0.05; ** = p < 0.01; *** = p < 0.001). Significant correlations were interpreted as follows: r_s_ = 0–0.10: negligible correlation; r_s_ = 0.10–0.39: weak correlation; r_s_ = 0.40–0.69: moderate correlation; r_s_ = 0.70–0.89: strong correlation; r_s_ = 0.90–1.00: very strong correlation^37^. Data for some parameters were also visually represented as scatter plots with linear regression line.

### Study 2

From each of eight older animals in the herd, one hematologic record at the youngest age (between 1 and 3 years), one record from middle age (between 4 and 6 years), and one record at the most advanced age (between 7 and 11 years) were compared with each other. Since these records were considered as paired samples, the individual parameters were determined using either a repeated measures ANOVA and a paired t-test with Bonferroni p value adjustment as post-hoc-test (if the Shapiro–Wilk test revealed p > 0.05), or a Friedman rank sum test, and Wilcoxon signed-rank test with continuity correction (if Shapiro–Wilk test revealed p < 0.05).

#### Albumin concentration

The available albumin values were presented as descriptive statistics (mean, standard deviation, minimum and maximum). Data were tested for normal distribution (Shapiro–Wilk test) and subsequent correlation analysis (Pearson’s linear correlation coefficient) was performed between albumin and HCT, hemoglobin, RBCs, MCV, MCH, and MCHC.

### Ethics statement

The animal facility at the Max Planck Institute for Multidisciplinary Sciences in Göttingen is registered in accordance with §11 para 1 TierSchG (Tierschutzgesetz der Bundesrepublik Deutschland, Animal Welfare Law of the Federal Republic of Germany) as documented by 39 20 00_2a) Si/rö , dated December 11, 2013 ("Erlaubnis, Wirbeltiere zur Versuchszwecken zu züchten und zu halten", "Permission to breed and to keep vertebrates for Experimental Purposes") by the Veterinär- und Verbraucherschutzamt für den Landkreis und die Stadt Göttingen (Veterinary and Consumer Protection Office for the district and city of Göttingen).

## Results

### Study 1

Hemoglobin (Hb), HCT, MCV, MCH, neutrophils, lymphocytes, NLR, PLR, and LMR showed significant changes in the evaluated hematologic records correlating to the increasing age of the animals.

#### Descriptive results

Mean, SD, median, lower and upper quartile as well as minimum and maximum of the individual parameters per year of life are displayed in Supplementary Table S3. In the numerical data, it became evident that the mean number of RBCs remained constant at approx. 12 × 10^12^/L for the entire study period, while size (MCV) and hemoglobin content (MCH) of the RBCs increased with age. Mean MCV was 32.7 fL in animals at one year of age and > 40 fL in animals older than 10 years; mean MCH increased from 10.3 pg in animals one year of age to almost 12 pg in animals older than eight years. These altered properties of RBCs were also reflected in increasing hemoglobin content and HCT with age. Mean Hb increased from approx. 130 g/L in animals aged one year to over 140 g/L in older animals (eight years and older).

In the leukogram, age-related changes were seen mainly in neutrophils and lymphocytes. While the percentages of neutrophils and lymphocytes in animals aged one year were 48.5% and 35.9%, respectively, the percentage of neutrophils increased with age and the percentage of lymphocytes decreased with age so that neutrophils were about 10% higher and lymphocytes about 10% lower in animals over eight years of age. In contrast, the absolute numbers of neutrophils varied from year to year. However, there was no evident increase in the absolute numbers [× 10^9^/L]. The situation was different for lymphocytes, where relative as well as absolute numbers revealed a clear decrease. While animals aged one year had a lymphocyte count of approx. 5 × 10^9^/L, animals over eight years had a lymphocyte count of only approx. 3 × 10^9^/L.

The ratios NLR, PLR, and LMR revealed broad ranges in some cases, but NLR and PLR increased numerically, while LMR decreased numerically with increasing age.

#### Correlation of hematologic parameters and age in days

**Figure 2:**
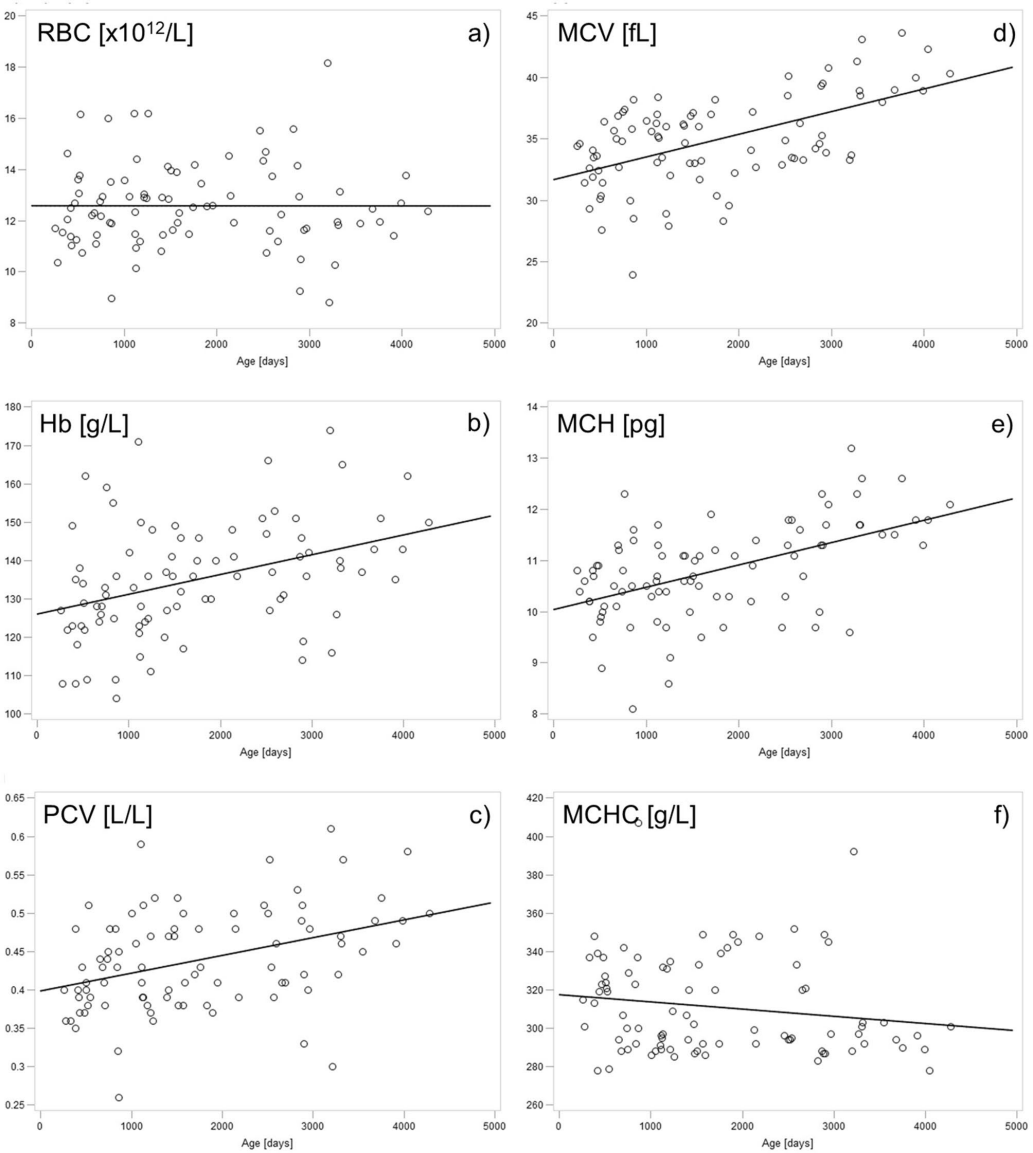
Study 1: Scatter plots and linear regression of hematologic parameters of the RBCs with age progression. While the RBC count (**a**) remained constant, Hb (hemoglobin) (**b**), HCT (**c**), MCV (**d**), and MCH (**e**) increased with age. MCHC (**f**), on the other hand, seems to decrease with age, but this was not a statistically significant finding.

**Figure 3:**
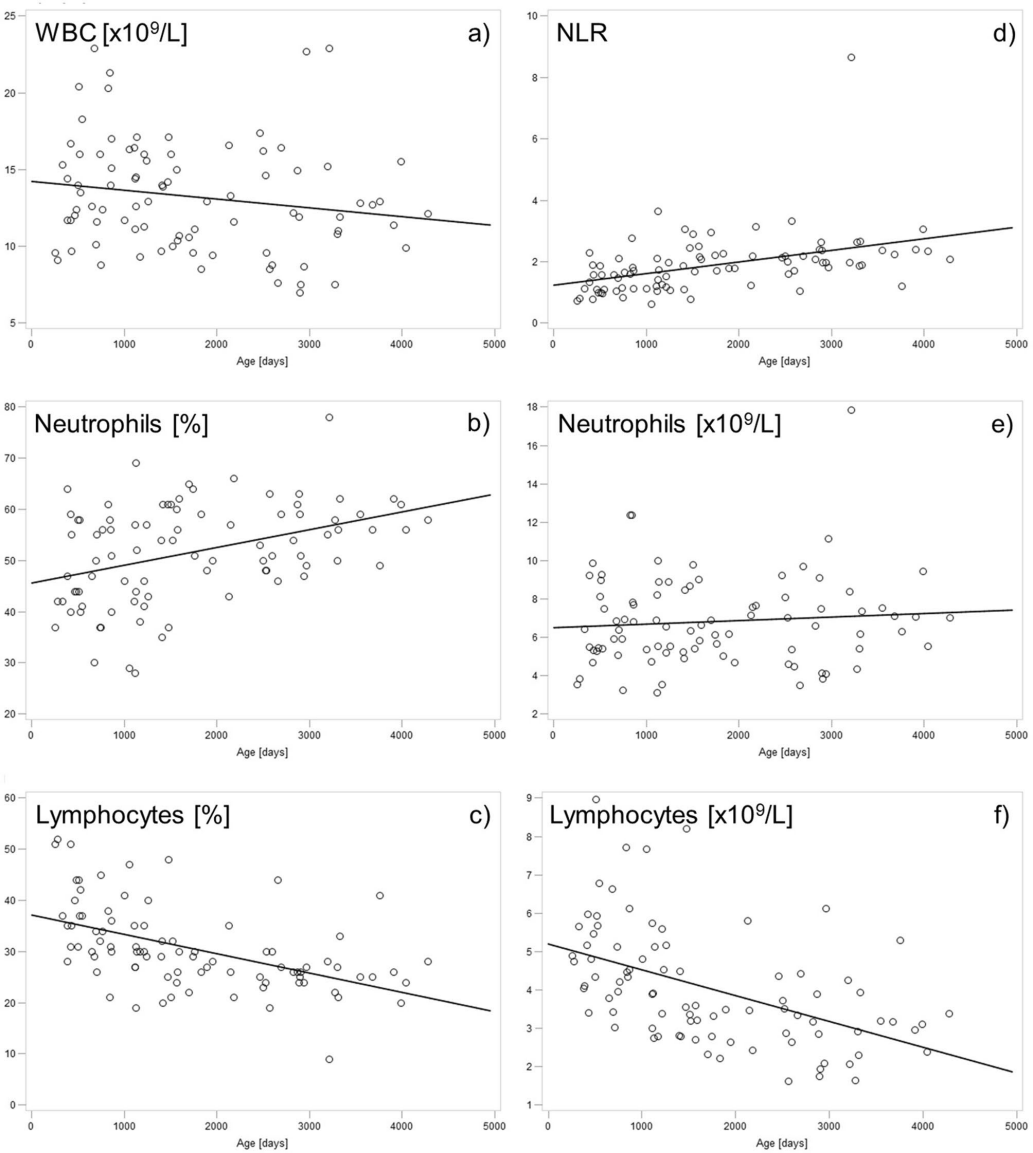
Study 1: Scatter plots and linear regression of hematologic parameters of the white blood cells during age progression. The WBC count (**a**) appears to decrease slightly with age, but this was not found to be statistically significant. Both the percentage and the absolute number of lymphocytes (**c**, **f**) decreased with age, whereas the percentage of neutrophils (**b**) increased, which is not reflected in the absolute number of neutrophils (**e**). The decrease in lymphocytes and increase in neutrophils are also reflected by an increase in the NLR (neutrophil-to-lymphocyte ratio) (**d**) with age.

Spearman´s rank correlation coefficient between age in days and the individual hematologic parameters showed a significant moderate positive correlation with Hb, MCV, MCH, neutrophils [%], PLR, and NLR, and a significant weak positive correlation with HCT. Negative correlations with age in days were seen in LMR (weak), and lymphocytes in both [%] (moderate) and [× 10^9^/L] (moderate). The exact results of the correlation analysis are displayed in Table 1 and in Figs. 2 and 3.

**Table 1:** Results of study 1: Spearman´s rank correlation coefficient (r_s_) and the corresponding p-values. Each hematologic parameter was correlated with the age of the animal in days for each sample. For this purpose, several records of different ages were used from each of the 21 female alpacas (n = 87, except for LMR where n = 68). An overview of records per single animal is provided in supplementary Table S1. NLR, neutrophil-to-lymphocyte ratio; PLR, platelet-to-lymphocyte ratio; LMR, lymphocyte-to-monocyte ratio.

	r_s_	p
WBC count [× 10^9^/L]	−0.20	0.066
RBC count [× 10^12^/L]	0.03	0.773
Hemoglobin [g/L]	**0.41**	** < 0.001**
HCT [L/L]	**0.39**	** < 0.001**
MCV [fL]	**0.49**	** < 0.001**
MCH [pg]	**0.49**	** < 0.001**
MCHC [g/L]	−0.16	0.133
Platelets [× 10^9^/L]	0.04	0.692
Neutrophils [%]	**0.40**	** < 0.001**
Lymphocytes [%]	**−0.60**	** < 0.001**
Monocytes [%]	−0.08	0.486
Eosinophils [%]	0.15	0.160
Basophils [%]	0.02	0.878
Neutrophils [× 10^9^/L]	0.07	0.516
Lymphocytes [× 10^9^/L]	**−0.57**	** < 0.001**
Monocytes [× 10^9^/L]	−0.11	0.318
Eosinophils [× 10^9^/L]	0.05	0.624
Basophils [× 10^9^/L]	0.02	0.841
NLR	**0.57**	** < 0.001**
PLR	**0.43**	** < 0.001**
LMR	**−0.31**	** < 0.01**

Significant values are in bold.

### Study 2

#### Comparison of eight animals at young, middle, and advanced age

In a paired comparison of hematologic records of eight animals each at different ages (young [1.5 ± 0.8 years], middle [4.6 ± 0.7 years], and old [9.0 ± 1.7 years]), differences due to age were detected for Hb, HCT, MCV, MCH, MCHC, lymphocytes [%], and [× 10^9^/L], eosinophils [%], and [× 10^9^/L] as well as neutrophils [× 10^9^/L] in the repeated measures ANOVA or the Friedman rank sum test.

Comparison of young vs. old revealed significantly higher HCT and MCV and significantly lower MCHC in old alpacas. Although a decrease in lymphocytes was numerically detected, this did not prove to be statistically significant. Comparison of young vs. middle revealed significantly higher eosinophils [× 10^9^/L] and [%], and significantly lower neutrophils [%] in alpacas at middle age. Comparison of middle vs. old revealed significantly higher Hb, HCT, MCV, neutrophils [× 10^9^/L], and monocytes [× 10^9^/L] as well as decreased MCHC in alpacas at advanced age. The exact results of study 2 are displayed in Table 2.

**Table 2:** Results of study 2. Data for eight alpacas at three different stages of life (young [1.5 ± 0.8 years], middle [4.6 ± 0.7 years], old [9.0 ± 1.7 years]). P-values for analysis of variance or Friedman rank sum test^a^. The post-hoc test was either the paired t-test with Bonferroni correction, or the Wilcoxon rank sum test with continuity correction^b^. The descriptive statistics for these animals can be found in Supplementary Table S4. NLR, neutrophil-to-lymphocyte ratio; PLR, Platelet-to-lymphocyte ratio; LMR, lymphocyte-to-monocyte ratio.

	ANOVA/Friedman	Young/middle	Young/old	Middle/old
WBC count [× 10^9^/L]	0.050	0.084	1.000	0.315
RBC count [× 10^12^/L]	0.127	1.000	0.340	0.280
Hemoglobin [g/L]	**0.011**	1.000	0.087	**0.013**
HCT [L/L]	** < 0.01**^a^	1.000^b^	** < 0.01**^b^	** < 0.01**
MCV [fL]	** < 0.001**	1.000	** < 0.01**	** < 0.01**
MCH [pg]	0.202	1.000	0.400	0.170
MCHC [g/L]	** < 0.01**^a^	0.344^b^	** < 0.001**	** < 0.01**^b^
Platelets [× 10^9^/L]	0.204	0.500	1.000	0.570
Neutrophils [%]	0.124	**0.017**	1.000	0.357
Lymphocytes [%]	**0.012**	0.160	0.074	0.409
Monocytes [%]	0.094^a^	0.380^b^	1.000^b^	0.100^b^
Eosinophils [%]	** < 0.001**	** < 0.01**	0.102	0.320
Neutrophils [× 10^9^/L]	**0.021**	0.093	1.000	**0.036**
Lymphocytes [× 10^9^/L]	**0.029**	0.170	0.150	1.000
Monocytes [×10^9^/L]	0.072^a^	0.338^b^	1.000^b^	**0.029**^b^
Eosinophils [×10^9^/L]	**0.012**	** < 0.01**	0.147	1.000
NLR	0.072^a^	0.703^b^	0.062^b^	0.114^b^
PLR	0.837	1.000	1.000	1.000
LMR	0.472^a^	1.000	0.440^b^	0.490^b^

Significant values are in bold.

#### Albumin concentration

Albumin was 37.8 ± 3.9 g/L (mean ± SD) for all samples tested, with a range of 27.2–47.5 g/L. Mild hypoalbuminemia and mild hyperalbuminemia were observed at one examination time and six examination times, respectively, compared to the RI from Dawson et al. ^36^. There was a significant weak positive correlation between albumin and HCT (r = 0.34; p = 0.002).

## Discussion

The retrospective evaluation of the hematologic findings of the alpaca herd revealed significant differences in several parameters with respect to the age of the animals. As the data were obtained from a standardized experimental herd that was kept under constant veterinary supervision and data were carefully screened to include only records from clinically healthy animals, we assume that these changes may reflect physiologic processes caused by the aging of the animals.

Among the RBC parameters, the RBC count did not show any age-related differences in either study. However, when comparing young and adult alpacas, Husakova et al. ^27^ found a significant decrease in the RBC count. An effect on HCT and Hb due to different hydration status of the animals seems unlikely, as albumin concentrations were within the RI in most samples. Hb, HCT, and MCV increased with age in both studies, which could be explained by the fact that older alpacas have larger RBCs, resulting in a higher total RBC volume (= HCT) than younger animals, despite an unchanged RBC count. An age dependence of the MCV is also known in other species and humans^39–41^.

A significant increase in MCH was only seen in study 1. In addition, study 2 showed a simultaneous significant decrease in MCHC, which was only numerically observed in study 1. An increase in MCH is the cause of an increase in Hb at older ages. However, RBC volume (MCV) seemed to increase more than hemoglobin content, as indicated by a decrease in MCHC. There appear to be species differences in MCHC with age. For horses and cattle, MCHC has been reported to increase with age^40,42^; in sheep and goats, MCHC fluctuates with age^41^.

When the mean of all records was compared with previously published RI for hematology in alpacas from the literature^19,20,23–27^, it is noticeable that the MCHC was significantly lower and that the mean of the HCT and the mean of MCV were significantly higher than these RI. An exception is the study by Stanitznig et al.^28^ which also used flow cytometry. In their study, the RI were higher for HCT and MCV and lower for MCHC than in the other studies mentioned above. When the mean values for all animals in our studies were compared with the RI of Stanitznig et al.^28^ for MCV, HCT, and MCHC, there were no deviations. The specific RBC morphology of camelids could be the reason for the different data in the RI. Weiser et al.^14^ as well as Vap and Bohn^8^ reported different results for HCT and MCHC after hematologic measurements in analyzers, which could be attributed to shape and size of the camelid RBCs. In Stanitznig et al.^28^ and other studies on RI for SACs, the exact analyzer was not always specified. In addition to the ADVIA 2120 ^20^, previous studies used different Coulter Counters (model ZF; Coulter Electronics Pty Ltd; North Ryde, Australia^23^; model ZBI, Coulter Electronics Inc, Hialeah, FL, USA^43^), or other devices like BC-2800 Vet auto hematology analyzer (Mindray Medical International Limited, Shenzhen, China^27^). However, there are no studies that have compared these devices with each other.

An increase in MCV in adults compared to crias has also been described by Hengrave-Burri et al.^19^ and Husakova et al.^27^. Husakova et al. also reported higher MCH in adults^21^. When comparing crias and adult alpacas, there was no increase in Hb or HCT^19,27^. According to Hengrave Burri et al., adult females even have a lower Hb than crias^19^; according to Husakova et al., crias have a higher RBC count^27^. The seasonal differences in RBCs, Hb, MCH, MCHC, WBCs, neutrophils, and monocytes described by Husakova et al.^27^ probably do not play a major role in our data, as most of the samples were collected in summer.

When considering young animals, variations within the first few days of life must also be taken into account, but data on hematologic changes in alpaca crias are scarce. Felton’s study found a drop in Hb within the first three days of newborn alpaca crias^44^. However, studies in other species must be considered for further information. A decrease in Hb has also been described in newborn bovine calves^45–47^. Mohri et al. found a decrease in both Hb and HCT up to 28 days of age and a significant increase thereafter^47^. MCV and MCH of calves decreased significantly within the first 56 and 42 days, respectively, and showed a significant increase thereafter. The RBC count of the calves did not decrease, but increased from the first day of life. Dynamic changes in the hematologic values during the first weeks of life have also been reported in other species such as dogs^48,49^.

Within the parameters of the WBCs, we found a decrease in relative and absolute numbers of lymphocytes with age in both studies. In neutrophils, we found an increase in relative numbers with age in study 1, although the clinically relevant absolute number of neutrophils did not change. Nonetheless, in study 2, a decrease in relative numbers of neutrophils between the young and the middle group and a subsequent increase in absolute numbers of neutrophils between the middle and the old group was observed. Eosinophils increased in study 2 from young to middle age. In study 1, this trend was not observed, presumably due to a slight decrease from the middle to the old group preventing a linear correlation over all years. Higher absolute numbers of neutrophils in adults compared to crias have been described by Husakova et al.^27^. Hengrave-Burri et al. determined no difference in this respect^19^. In other species, there are hints that older animals have higher neutrophil counts than young animals^18^, but there are also other reports that have not found any difference^50,51^. A decrease in lymphocytes from young to adult animals was also found by Hengrave-Burri et al.^19^. Husakova et al., in contrast, reported a significant increase^27^. For other species like cats^50^, dogs^52^, deer^18^, and horses^53^, a decrease in lymphocytes with age is also reported. The change in the lymphocyte counts might be associated with changes in subpopulations. Fujiwara et al. found a decrease in both naïve CD4 + and naïve CD8 + cells as well as decreased activity of T-cell related cytokines in older dogs. They assumed that this might be related to age-related immunosenescence (deterioration of the immune system with age)^54^.

However, the changes in neutrophil and lymphocyte counts seem to apply only to animals after an age of 2–3 months. In calves, regularly examined within the first months of life, it is the other way round; it could be shown that neutrophils first decrease during the first weeks of life, whereas lymphocytes increase^47^. On the basis of our data, it remains unclear if such changes also apply to alpaca crias. Further investigations in this respect are certainly necessary.

No definitive statement can be made about band neutrophils in alpacas in our study. It is noteworthy that only one animal was found to have a single band neutrophil, although these are occasionally found in healthy alpacas^55^. Nevertheless, similar results are reported by Dawson et al.^20^: In a study of hematologic RI in alpacas, 65 healthy animals were subjected to both differential blood counts using the ADVIA 2120 and microscopic differentiation of 100 cells in the blood smear. No band neutrophils were found by either method.

Our investigations do not allow a clear statement about the influence of age on eosinophils. While no age-dependent effect on eosinophils could be detected in study 1, a significant increase in eosinophils from the age groups young to middle was found in study 2 in both the eosinophil proportion and the eosinophil count. Hengrave-Burri et al. and Husakova et al. also found significantly higher absolute eosinophils in adult alpacas than in crias^19,27^. Nonetheless, this was independent of fecal egg excretion^19^.

The different results for relative and absolute WBC distributions clearly show that absolute numbers should be used to interpret hematologic findings, as percentages on the leukogram give only a relative picture and should be used in conjunction with the total WBC count.

To date, little is known about NLR, PLR, and LMR in alpacas. NLR has been studied more extensively in other species and is associated with acute and chronic stress^56–59^. Montes et al. found the range for NLR in 30 alpacas to be 0.2–1.1 ^60^, and within the RI published by Hajduk, the NLR was reported to be 0.5–2.9 ^23^. However, these data were not differentiated according to the age of the animals. In study 1, we found an increase in NLR with advancing age. The NLR for all 87 data sets in our study was 1.89 ± 0.99, which is consistent with Hajduk's data, but when looking at individual years, the mean NLR varied from 0.85 in animals < 1 year of age to 3.41 in animals 7 years of age. The increase in NLR did not appear to be linear, but occurred mainly in the first three years of life (Supplementary Table S3). From the fourth year of life, only fluctuations, but no increase in NLR could be observed in study 1. Nevertheless, this was not reflected in study 2, which could be due to the fact that records from the age of one to three years of age were included for the "young" time point. An increase in NLR with age has also been reported in horses^53^.

To the best of our knowledge, there is no literature on LMR and PLR in alpacas so far. LMR is used in human medicine as a prognostic marker for several neoplasms^61^. In dogs and cats, it has recently been shown that a low LMR is an indicator of poor prognosis in lymphoma^62,63^, which is also common in alpacas^4,64,65^. In human medicine, the PLR is also used as a marker for inflammation or neoplasia. A high PLR is associated with a poorer prognosis^66,67^; this parameter is also used in veterinary medicine^68^. Although little is known about the usefulness of these lymphocyte ratios in alpacas, future studies using these parameters should consider age dependence.

Our data confirm the previously reported changes of hematologic parameters with age. In addition, we were also able to show differences within the group of adult alpacas. Interestingly, these changes appear to be most pronounced at older ages. The animals studied here had a maximum age of 11 years, but alpacas can reach an age of 20 years and older^69^. The extent to which hematologic parameters change in animals over 11 years of age is therefore not represented in our data.

### Limitations

As study 1 and study 2 are retrospective evaluations of a single herd over a long period of time, some limitations must be taken into account when interpreting the results:

- The number of samples in our preliminary study was limited, and the number of animals per group varied considerably in study 1.The data given should not be considered as RI, but are only used to follow the progression with age. A prospective study with a larger number of animals would be needed to validate the statements.

- The samples were not tested directly as they were sent to an external laboratory. Therefore, possible changes due to storage time (up to 3 days, median: 1 day) as described for other species^70^ cannot be completely excluded. In a study where human blood was stored at room temperature for 72 h after collection and then measured on the ADVIA 2120i, there were statistically significant increases in RBC, HCT, MCV, and statistically significant decreases in WBC, MCH, MCHC, and platelets^71^. However, as the time delay affected all samples, we consider this to have a minor effect on our results.

- HCT was determined automatically by calculation and not by centrifugation, which may explain the sometimes high values compared to the RI. As all measurement procedures were standardized, we believe that the changes shown here with increasing age are plausible. Nevertheless, the absolute values should be used with caution when comparing with other values. Automated measurement of camelid RBCs is generally limited; unlike other species, RBCs do not sphere in the diluent of the ADVIA 2120 ^34^.

- Microscopic verification of differentiation was only available for one third of the records. However, studies by Dawson et al.^20^ showed a close association between the results of the ADVIA 2120 and WBC differentiation when using a microscope in alpacas.

- Some of the animals were hybrids. As alpacas and llamas can be crossed and produce fertile offspring, it is likely that there is an unknown proportion of hybrids in the German alpaca population, as many farms keep both species. We decided to include these animals in the evaluation, as the data did not show any numerical differences from the other animals. A statistical comparison between the hybrids and the other animals was not possible due to the small and irregular number of records of these animals. To our knowledge, there is no information in the literature on the hematology of alpaca hybrids.

## Conclusion and outlook

The results of our studies show that some hematologic parameters in female alpacas change with age, which must be taken into account when interpreting hematologic findings. While age had no effect on the RBC count, there were shifts in RBC volume and -hemoglobin content, as reflected by the increasing hemoglobin and hematocrit levels. Among WBCs, there was a decrease in lymphocyte count and an increase in neutrophil count, resulting in age-related changes in the lymphocyte ratios. In addition to age-related changes, differences in sex, season, and environment should also be taken into account^27,72,73^. These influences can be largely excluded in our studies because all alpacas were females, housed in the same location, and sampled mainly in summer. The reviewed studies in other species suggest that hematologic findings can be used as good prognostic markers. Further research into lymphocyte ratios in alpacas is needed. In addition to the described hematologic changes with age, age-related changes in blood cell composition and function should be characterized in more detail, as in other species^54,74^. The clinical relevance of our results should be verified in further studies related to the clinical parameters of the animals.

### Supplementary Information

Supplementary Information.

## Data Availability

The original contributions presented in the study are included in the article/supplementary material. Further inquiries can be directed to the corresponding author/s.

## Abbreviations

BW: Bodyweight
Hb: Hemoglobin
HCT: Hematocrit
LMR: Lymphocyte-to-monocyte ratio
MCH: Mean corpuscular hemoglobin
MCHC: Mean corpuscular hemoglobin concentration
MCV: Mean corpuscular volume
NLR: Neutrophil-to-lymphocyte ratio
PLR: Platelet-to-lymphocyte ratio
RBC: Red blood cell
RI: Reference interval
SACs: South American camelids
WBC: White blood cell
